# Decoding the Role of Interleukin-30 in the Crosstalk between Cancer and Myeloid Cells

**DOI:** 10.3390/cells9030615

**Published:** 2020-03-04

**Authors:** Emma Di Carlo

**Affiliations:** 1 Department of Medicine and Sciences of Aging, “G. d’Annunzio” University of Chieti-Pescara, 66100 Chieti, Italy; edicarlo@unich.it; Tel.: +39-0871-541540; 2 Anatomic Pathology and Immuno-Oncology Unit, Center for Advanced Studies and Technology (CAST), “G. d’Annunzio” University of Chieti-Pescara, 66100 Chieti, Italy

**Keywords:** tumor microenvironment, myeloid cells, interleukin-30, metastasis, cancer stem-like cells, anti-tumor immune response

## Abstract

In the last few years, a new actor hit the scene of the tumor microenvironment, the p28 subunit of interleukin (IL)-27, known as IL-30. Its molecular structure allows it to function as an autonomous cytokine and, alternatively, to pair with other subunits to form heterodimeric complexes and enables it to play different, and not fully elucidated, roles in immunity. However, data from the experimental models and clinical samples, suggest IL-30′s engagement in the relationship between cancer and myeloid cells, which fosters the tumor microenvironment and the cancer stem cell niche, boosting the disease progression. Activated myeloid cells are the primary cellular source and one of the targets of IL-30, which can also be produced by cancer cells, especially, in aggressive tumors, as observed in the breast and prostate. This review briefly reports on the immunobiology of IL-30 and related cytokines, by comparing mouse and human counterparts, and then focuses on the mechanisms whereby IL-30 amplifies intratumoral myeloid cell infiltrate and triggers a vicious cycle that worsens immunosuppression in the tumor microenvironment (TME) and constitutes a real threat for a successful immunotherapeutic strategy.

## 1. Introduction

Tumor-associated immune cells and their crosstalk with cancer and the components of its microenvironment dramatically affect tumor behavior and patient outcome [1,2]. The concept of cancer immunoediting, represents the dynamic of this interaction, which runs through the phases of elimination, equilibrium and escape, and explains how immunosuppression can overwhelm immunosurveillance [3], eliciting the development of immunological-based approaches to cancer treatment [4,5].

Histopathological analysis of experimental and clinical tumors has revealed that, although there is great variability, according to the tumor type and clinical-pathological profile of each patient [5,6], virtually all types of immune cells, including different subsets of T and B lymphocytes, NK and innate lymphoid cells can be part of the tumor microenvironment (TME). However, myeloid cells are the most widely represented, since they might account for up to 50% of the neoplastic mass [6,7]. Through immunoregulatory molecules, chemoattractants and growth factors, the tumor recruits or modulates tissue–resident (derived from embryonic precursors in the yolk sac and fetal liver) and the circulating myeloid cells (originated from the bone marrow hematopoietic stem cells). These innate immunity cells become tumor-associated macrophages, dendritic cells (DCs), myeloid-derived suppressor cells and granulocytes, which may condition cancer, immune and stromal cell viability and function, and sustain an immunosuppressive environment [8,9]. This is accomplished by their release of reactive oxygen species (ROS), nitric oxide (NO), and ectoenzymes, which regulate the adenosine metabolism; expression of immune checkpoint molecules [10,11]; depletion of metabolites critical for lymphocyte functions [12,13]; and through their secretion of growth, angio- lymph-angiogenic factors and inflammatory mediators [13], that are crucial for tissue remodeling and tumor development.

Recent insights into this inflammatory milieu, specifically in breast (BC) and prostate (PC) cancers, have unveiled a role for the novel immunoregulatory mediator Interleukin(IL)-30/IL-27p28 [14] in the TME and in the intricate relationship between cancer and myeloid cells, which orchestrates tumor-promoting events with evident clinical implications [15,16].

## 2. IL-30 as a Self-Standing Cytokine or Cytokine-Like Subunit Paired with Soluble Receptor-Like Proteins to Form Different Heterodimeric Complexes

IL-30 was discovered in 2002, through a computational screen of expressed sequence tags (ESTs) to identify orphan homologs of the IL-6 and IL-12 family [14], and is also known as IL-27p28, according to its molecular mass of 28 kDa [14]. IL-30 constitutes the four-α-helix bundle (cytokine-like) subunit of the heterodimeric cytokine IL-27 [17]. Both in humans and in mice, IL-30 interacts via non-covalent bonds with the IL-27 β-subunit, EBI3, the (receptor-like) protein encoded by the Epstein Barr virus-induced gene 3, to form the bioactive IL-27 [18]. Genes encoding for homologs of IL-27 subunits have been identified in nearly 20 mammalian species [17].

### 2.1. IL-27 Involvement in Cancer-Myeloid Cell Crosstalk

Myeloid cell populations, including macrophages, monocytes, microglia, and DCs, activated by a variety of microbial and immune stimuli (TNF family members CD40 and CD137 [18,19], type I and type II IFNs, [20,21,22,23,24]) are the major cellular sources of IL-27, which can also be produced by neutrophils [25], plasma cells, endothelial and epithelial cells [26].

IL-27 receptor (R) consists of a ligand-binding chain, IL-27Rα (WSX-1, TCCR), expressed on DCs, monocytes, macrophages, neutrophils, mast cells, eosinophils, T, B, and NK cells [27], up-regulated after cellular activation, and the signal-transducing chain, gp130, which is expressed by virtually all cell types [28]. A variety of cancer cell types also express IL-27R and respond to IL-27, which can inhibit neoplastic cell proliferation, migration and invasion, and promote apoptotic cell death, revealing direct anti-tumor effects [29,30,31,32,33]. Binding of IL-27 to its receptor results in the activation of JAK-STAT signaling pathways and immunoregulatory functions [34].

As both source and target of IL-27, myeloid cells are undoubtedly involved in its dual anti- and pro-tumor activity [35], which would be contingent on the specific type of tumor [34]. In human non-small-cell lung cancer xenograft, IL-27 down-regulates cancer cell expression of stemness- and epithelial–mesenchymal transition (EMT)-related genes, but also re-educates intratumor myeloid cells to exert antitumor effects [36], which are abolished by myeloablation, thus, suggesting IL-27′s ability to bolster innate immune responses.

Indeed, IL-27 has a regulatory role on myeloid cells, since it has revealed both activating and suppressive effects [37].

IL-27 promotes differentiation of human monocytes into macrophages and enhances their production of pro-inflammatory cytokine, such as IL-6, TNF-α, MIP-1α, and MIP-1β [38,39]. In human monocytes, IL-27 also upregulates HLA-E, which, upon interacting with CD94/NKG2A [40], suppresses the NK cell functions and IFNγ release which, in turn, weakens the anti-tumor immunity [41]. IL-27 also triggers NK cell cytotoxicity through the upregulation of perforin and granule exocytosis [42], by contrast, it inhibits the activity of CD56^bright^ NK cell subsets [43].

IL-27–STAT3 axis has been reported to induce expression of programmed cell death 1 and 2 ligands (PD-L1/2) on the infiltrating macrophages, in adult T cell leukemia/lymphoma and diffuse large B-cell lymphoma microenvironments [44]. In human monocytes isolated from peripheral blood [45] and in neonatal macrophages, IL-27 also induces the expression of the immune suppressive enzyme indoleamine 2,3-dioxygenase (IDO), which displays regulatory functions on T cell proliferation, but also inhibits CD4^+^ T cell proliferation and induces IL-17 and IL-10 secretion [46]. Furthermore, IL-27 induces IDO and PD-L1 expression in human SKOV3, CAOV3, OC316 ovarian adenocarcinoma cells, PC3 prostate cancer cells, and A549 lung adenocarcinoma cells [45]. In ovarian cancer, neutrophil-derived IL-27 maintains the immunosuppressive phenotype of tumor-associated macrophages, by increasing ectonucleoside triphosphate diphosphohydrolase-1 (ENTPD1)/CD39 and PD-L1 expression, and IL-10 production [25]. Finally, IL-27 can exert [47] immunosuppressive activity on the myeloid cells, by boosting IL-10 production in Th1, Th2, Th17, Treg, and Tr1 cell subsets, which in turn suppress macrophage and DC production of inflammatory cytokines, in response to different stimuli, such as TNFα, IL-1, and multiple Toll-Like Receptor (TLR)s [48]. These findings suggest a context-dependent activation *versus* suppressive functions of IL-27 in innate immunity and highlight its homeostatic role in limiting macrophage activation through inflammatory cytokines.

In human DCs, IL-27 directly up-regulates B7 homolog 1 (B7-H1), i.e., PD-L1, decreases HLA restricted antigen presentation, and inhibits proliferation and cytokine production in allogeneic T cells [49,50]. Despite its inhibitory functions on both murine and human DCs, IL-27 has revealed immune-stimulatory properties on cord blood (CB) DCs obtained from the human neonate. In the specialized immune system of the newborn, IL-27 has shown to increase its own production and to promote migration and functions of CBDCs by increasing the transcription of *C-X-C motif chemokine 10 (CXCL10)*, chemokine receptor *CCR1*, *interferon regulatory factor 8 (IRF8)*, and genes involved in antigen presentation [51]. Activities of IL-27 and the outcome of the response to IL-27 are highly dependent on cell type, activation state, and microenvironmental context.

### 2.2. IL-30/CLF

In addition to its pairing with EBI3, which results in IL-27 production, IL-30 can associate, in both mouse and humans, with another soluble cytokine receptor, Cytokine-Like Factor 1 (CLF) encoded by the *cytokine receptor-like factor 1 (CRLF1)* gene, resulting in a bioactive heterodimer that can be secreted by activated DCs.

IL-30/CLF complex engages a tripartite receptor composed of IL6Rα, in addition to the IL-27R subunits gp130 and IL-27Rα, and promotes, in both mouse and humans, the activation of T and NK cells. In particular, IL-30/CLF induces STAT1 and STAT3 phosphorylation in CD4^+^ and CD8^+^ T cells and IL-17 and IL-10 production in CD4^+^ T cells, whereas it inhibits CD4^+^ T cell proliferation [52].

Although it is unable to affect cytotoxic activity in NK cells, IL-30/CLF has been shown to increase IL-12- and IL-2-induced IFNγ production and activation marker (CD54 and CD69) expression, suggesting its involvement in the cross-talk between DCs and NK cells [52].

IL-30/CLF has also been revealed to sustain murine plasmacytoma cell proliferation and B cell differentiation and to behave similar to IL-6 [53], but the lack of corroborating evidence in humans precludes hypothesizing any involvement in human pathology.

### 2.3. IL-30/IL-12p40

In the murine model, through genetic engineering, IL-30 has been coupled with the IL-12β subunit, IL-12p40, to form a heterodimeric complex that can inhibit STAT1 and STAT3 signaling, downstream of IL12Rβ1 and gp130 receptors, and can efficiently suppress T cell functions. In particular, IL-30/IL-12p40 has shown to inhibit autoreactive Th1 and Th17 and to promote Treg cell expansion, leading to the resolution of experimental autoimmune uveitis [54]. However, a natural human counterpart of this molecular complex has not been demonstrated.

### 2.4. EBI3, IL-35, and IL-39 Involvement in Cancer-Myeloid Cell Crosstalk

EBI3 is a secreted 34kDa glycoprotein, composed of 229 amino acids in human (and 228 in mice), encoded on human chromosome 19 (mouse chromosome 17) [17]. It is also structurally related to soluble IL-6Rα (sIL-6Rα) [55] and to the secreted p40 subunit of IL-12 and IL-23 [56], which lacks a membrane-anchoring motif [57].

Induced in B lymphocytes by the Epstein-Barr virus (EBV) infection, EBI3 has been found in EBV-associated tumors, nasopharyngeal carcinoma, and Hodgkin lymphoma to inhibit an effective antitumor immune response, independent of its association to IL-30 [58,59]. EBI3 has revealed growth-promoting activity in lung cancer [60] and in colorectal cancer, by stimulating cell proliferation, via the gp130/STAT3 axis, and by restraining tumor infiltrating granzyme B^+^ CTLs and IFNγ^+^ CTLs [61], thus, allowing the cancer to escape immune surveillance.

EBI3 can associate with other cytokine subunits, such as IL-12p35, to form IL-35, which can be produced in humans and mice, mainly by regulatory B and T lymphocytes [62], and is involved in autoimmunity and cancer [63]. Macrophages can also produce IL-35 and activate the JAK2–STAT6–GATA3 signaling axis in cancer cells, which reverses EMT and facilitates metastasis [64]. IL-35 is produced in human cancer tissues, such as large B cell lymphoma, nasopharyngeal carcinoma, and melanoma. It promotes myeloid cell accumulation in the TME and, thereby, fosters tumor angiogenesis and growth [65]. Finally, EBI3 can associate with IL-23p19, to form IL-39, which is secreted by the activated murine B cells that mediate lupus-like diseases in MRL/lpr mice [66], but a clear demonstration of a functional human counterpart is lacking [67] and, therefore, its possible involvement in cancer [68] remains unclear.

### 2.5. IL-30 Immunobiology in Man and Mouse

The *IL-30* gene located on chromosome 16 in humans, and chromosome 7 in mice, encodes respectively, a 243 and 234 amino acid polypeptide, corresponding to the mature proteins, with a calculated molar mass of 24.5 and 23.6 kDa. Human and mouse *IL-30* are 73% identical [14]. However, due to a difference in a single amino acid residue, which affects protein folding (a disulfide bond between two nearby cysteines, which would stabilize the protein structure), murine IL-30, but not the human counterpart, can be efficiently secreted [14] by activated antigen presenting cells [14], and can exert immunoregulatory functions [52,69,70,71]. Although human IL-30 essentially depends on assembly with a β-subunit for secretion, substitution of a single amino acid residue, and other molecular mechanisms can affect protein folding and regulate its secretion competence. It has been reported that polymorphisms in the human *IL-30* gene can affect the regions identified to be important for its assembly-induced folding [72], which could lead to extracellular protein secretion with functional implications. Recent studies [73,74] suggest that common polymorphisms in the *IL-30* gene are linked to cancer risk and might contribute to the progression of cancers and chronic inflammatory diseases in humans.

Like the other members of the IL-6 family of cytokines, IL-30 has three potential receptor binding sites [75,76]. It binds EBI3 through site I, WSX-1 through site II, while gp130 recruitment is mediated by site III [66]. The binding of IL-30 to a gp130/WSX-1 heterodimer or a gp130 homodimer is selective and controlled by a molecular switch induced by EBI3 or IL-6R, respectively. IL-30 has functions unrelated to its capability to form a complex with EBI3. In both mice and humans, IL-30/IL-6R activates signal transduction, solely via the β-receptor chain gp130, with subsequent STAT1 and STAT3 phosphorylation, without the need of WSX-1. In addition, IL-30 can also form a biologically active complex with sIL-6R, both in mice and in humans [69,77], which implies that IL-30 trans-signaling is possible on cells lacking membrane-bound IL-6R [69], a mechanism that enlarges the spectrum of IL-30 responsive cells to virtually all cells of the body, in analogy to IL-6. Such as for the best known and longer studied homologue cytokine IL-6 [78,79], a prominent role is emerging for IL-30 in tumorigenesis, given its ability in shaping the TME and cancer stem cell niche, in which myeloid cells are critically involved.

## 3. IL-30 promotes the Expression of Myeloid Cell Growth and Chemotactic Factors in Cancer Cells

Produced by cancer and myeloid cells that infiltrate the tumor and draining lymph nodes (LN), IL-30 is emerging as a potential tumor growth factor, as found in BC [16] and PC [15,80,81], and is a regulator of genes involved in inflammation, immune-suppression and metastasis.

**Figure 1:**
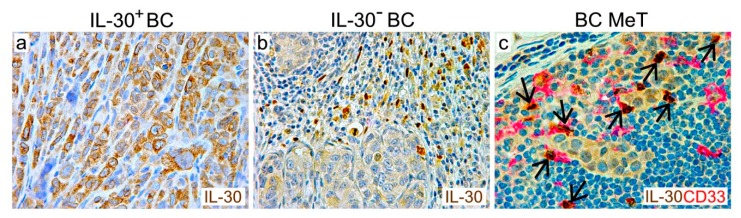
Expression of IL-30 in breast cancer and lymph node metastasis (MeT). Immunohistochemistry reveals the expression of IL-30 (in brown; ab118910, Abcam) in cancer cells of a Triple Negative breast cancer (BC) (**a**), in leukocytes infiltrating an HER2+ BC (**b**), and in both metastatic and myeloid cells (red), indicated by arrows (anti-CD33 antibody, clone PWS44; Leica Biosystems), in the lymph node draining this tumor (**c**). (Magnification: ×630).

Murine IL-30 has been shown to suppress the anti-tumor effects of IL-27, and to reduce the survival of colon cancer-bearing mice [70]. In vitro studies have revealed that, in both murine and human BC and PC cells, endowed with IL-6Rα and gp130, IL-30 treatment not only stimulates proliferation, but also activates a cancer progression program, including the production of myeloid cell proliferation and chemotactic factors [16,81], such as colony stimulating factor 1, CSF1 [82], chemokine C-X-C motif ligand 1 and 2, CXCL1, CXCL2 [83], IL-1β, IL-8 [84], and IL-6 [85]. Recruited at the tumor site, myeloid cells, specifically F4/80^+^ and CD11b^+^GR-1^+^ cells in mice, in turn express IL-30, thus, bolstering tumor growth and immune escape mechanisms. To this regard, IL-30 overproduction in the TME has shown to increase PD-L1 expression, not only in cancer cells, but also in tumor- and draining-LN-infiltrating immune cells [81]. Recent analyses of clinical BC and PC samples [15,16] revealed the expression of IL-30 in cancer cells and in tumor- or draining-LN-infiltrating leukocytes, mostly CD68^+^ macrophages, CD33^+^CD11b^+^ myeloid cells, and CD14^+^ monocytes, whereas it was absent in the normal tissue counterparts. IL-30 expression occurs, in particular, in poorly differentiated, high-grade and stage PC [15,80], which is characterized by intratumoral myeloid cells that suppress T-cell activity [86,87]. In breast cancer, IL-30 production in cancer and infiltrating leukocytes is associated with Triple Negative (TN) (which lacks estrogen- and progesterone-receptor expression and do not overexpress human epidermal growth factor receptor 2, HER2) and HER2+ molecular subtypes [16] (Figure 1a–c). In myeloid cells infiltrating primary tumors or draining LNs, IL-30 expression increases with disease stage and correlates with recurrence. Indeed, IL-30 expression by myeloid cells in breast cancer draining-LNs has been identified as an independent predictor of poor clinical outcome [16].

## 4. Tumor- and Myeloid Cell-Derived IL-30 Contributes to the Maintenance of the Cancer Stem Cell Niche

**Figure 2:**
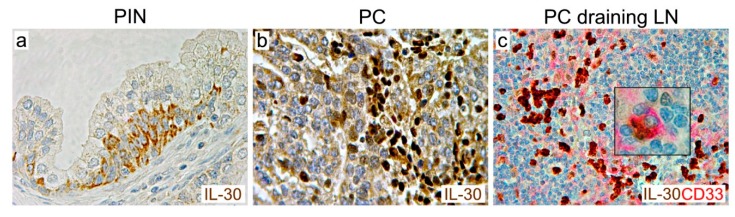
Expression of IL-30 in prostatic intraepithelial neoplasia, high-grade prostate cancer and myeloid cells of a cancer draining-lymph node. Immunohistochemistry reveals the expression of IL-30 (in brown; ab118910, Abcam) in basal stem-like cells of prostatic intraepithelial neoplasia (PIN) (**a**), in both cancer cells (faint) and leukocytes (strong) that infiltrate high grade (Gleason 8: 4 + 4) prostate cancer (PC) (**b**), and in most of the myeloid cells (in red; anti-CD33 antibody, clone PWS44; Leica Biosystems) in the lymph node that drains this tumor (**c**), as better shown by the inset. (Magnification: a, b: ×630; c: ×400; inset in c: ×1000).

Expressed in most of the high-grade and stage PCs, IL-30 has been recently found to be expressed by the rare CD133^+^ PC stem-like cells (SLCs) that are located in the basal layer of prostatic intraepithelial neoplasia (PIN) in humans [81] (Figure 2a–c). IL30 has also been detected in PC–SLCs isolated from PIN that spontaneously develop in TRAMP mice (hemizygous for the rat probasin-SV40gp6 large T antigen transgene, in a C57BL/6J background [88]), that recapitulate critical features of the human disease [89].

These murine PC–SLCs have a Sca-1^+^CD133^+^CD44^hi^α2β1^hi^CD49f^+^ phenotype [90], lack CD45 and CD31 markers [91], lack the androgen receptor and synaptophysin [92], and constitutively produce and release IL-30 [81], but they do not produce EBI3 or the IL-27 heterodimer [81]. Silencing or knocking out of *IL-30* in PIN-SCs, by using short hairpin (sh) RNA constructs that target the *IL-30* gene, or by Clustered Regularly Interspaced Short Palindromic Repeats (CRISPR)/Cas9 genome editing, respectively, causes a substantial reduction in their proliferation, colony-forming, and sphere-forming abilities and reveals the autocrine function of the cytokine [91].

When orthotopically implanted in congenic C57BL/6J mice, PC–SLCs allowed an in-depth investigation of the impact of *IL-30* overproduction or silencing in a fully immune-competent TME. This murine model provided evidence that besides driving PC–SLC self-renewal and metastatization, IL-30 has an impact on the immuno-molecular profile of PC, and on the recruitment and function of myeloid cell populations, as stated in the findings below.

**Figure 3:**
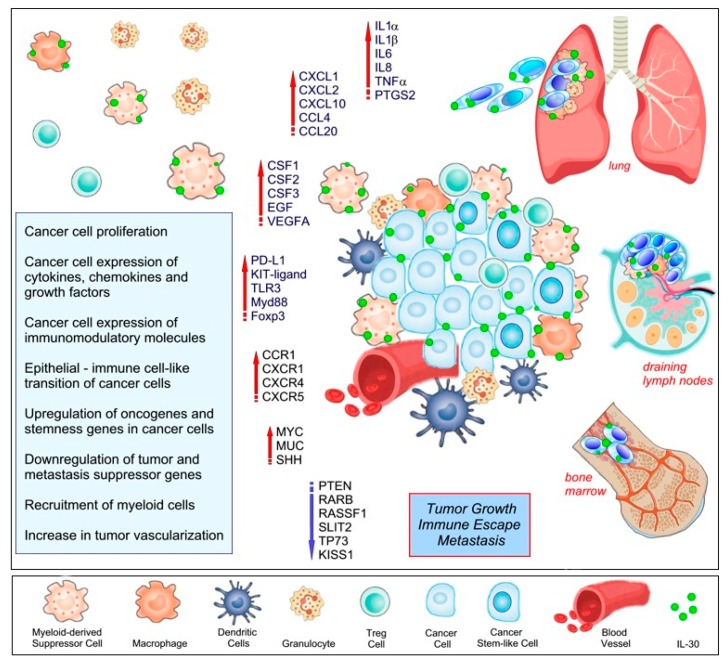
Effects of IL-30 expression by cancer and myeloid cells in the tumor microenvironment. On the left side, the effects of IL-30 on cancer cells and on the composition of immune cells infiltrating the tumor are listed. On the right side, the effects of IL-30 on tumor behavior are represented.

- IL-30 boosts PC–SLC expression of the KIT-ligand, TLR3, Myeloid differentiation primary response 88 (Myd88), Foxp3, and CD274/PD-L1 [82], which counteract the host’s anti-tumor immune response, and expression of chemokine C-C motif ligands 4 (CCL4), CSF2, CSF3, C-X-C motif ligand 1 (CXCL1) and 2 (CXCL2), prostaglandin-endoperoxide synthase 2 (PTGS2), also known as cyclooxygenase-2 (COX2), which along with IL-1β, IL-6, and TNFα [81], promote myeloid cell accumulation and suppressive functions [87]. In fact, the fast-growing tumors that develop from IL-30-overexpressing PC–SLCs, show a prominent myeloid cell infiltrate that mainly consists of F4/80^+^ macrophages, CD11b^+^ myeloid cells, and Ly-6G^+^ granulocytes, which along with tumor progression is hampered by IL-30 knockdown [81];
- IL-30 promotes ‘epithelial–immune cell-like transition’ [93] of PC–SLCs, mainly via STAT1/STAT3 pathways, through induction/up-regulation of expression of the chemokine receptors C-C chemokine receptor type 1 (CCR1), C-X-C motif chemokine receptor 1, 4, and 5 (CXCR1, CXCR4, and CXCR5). Specifically, IL-30 promotes PC–SLC spread to the bone marrow, by boosting local production of CXCL13, and cancer cell expression of CXCR5, which is suppressed by IL-30-silencing [81]. IL-30′s involvement in bone marrow colonization of PC–SLCs suggest that targeting of this cytokine can impact on bone metastasis, which is a crucial step in cancer progression and a leading cause of worsening outcome.
- IL-30 overproduction by PC–SLCs also promotes lung metastasis, involving the CXCR4/CXCL12 axis, since it induces CXCR4 expression on PC–SLCs and promotes their migration towards CXCL12^+^ pleural covering and bronchiolar walls. More than 80% of mice bearing IL-30 overexpressing tumors developed lung metastasis, *versus* 46–52% of mice bearing control tumors. Of note, once again, myeloid cells are a critical component of IL-30 conditioned microenvironment, since lung metastasis developed from IL-30 overexpressing tumors reveal a content of F4/80^+^, CD11b^+^, and Ly-6G^+^ cells that are significantly higher than the controls, in association with a prominent metastatic cell proliferation and vascularization [81]. Figure 3 summarizes the effects of IL-30 expression by cancer and myeloid cells on the TME, antitumor immune response, and tumor behavior.

It has been recently demonstrated that targeting IL-30 signaling, both in PC–SLCs and host environment, synergistically inhibits PC growth, reduces lung metastasis, and improves survival [91].

In *IL-30* conditional knockout (KO) mice (*EIIa-p28^f/f^*) [94], the lack of IL-30 production by the host leukocytes, essentially myeloid cells that are its main source, not only prevents expansion of CD4^+^CD25^hi^Foxp3^+^ Tregs and IL-10 production in both spleen and TME, following PC–SLC engraftment, but also favors the intratumoral influx of perforin^+^ cytotoxic T lymphocytes (CTLs), with a moderate cancer cell apoptosis. In the spleen of *IL-30*KO mice bearing tumors, the macrophage and DC networks, in contrast to their wild type counterparts, clearly expressed IL-12 and IFNγ. The absence of IL-30 in the host environment might slow down tumor growth by preventing Treg expansion and immunosuppressive functions, and by skewing the cytokine milieu towards a Th1-type, IFNγ- and IL-12-driven, immune response.

In wild type mice, *IL-30*-silencing in PC–SLCs, markedly prevents intratumoral infiltration of immunosuppressive IDO^+^CD11b^+^Gr-1^+^ myeloid cells [95], hinders cancer proliferation and vascularization, and reduces lung metastatization. The resulting inhibition of tumor progression, that was greater than that observed in *IL-30*KO mice bearing wild type tumors, reveals the consequences of IL-30 targeting in CSCs, which interrupts the IL-30 autocrine and paracrine loops.

When *IL-30* silenced PC–SLCs were implanted in IL-30KO mice, the lack of immunosuppressive IDO^+^ myeloid cells in the TME fostered intratumoral enrichment of FasL^+^ TRAIL^+^ CTLs, mainly intratumoral cytotoxic granule-associated RNA binding protein 1 (TIA-1)^+^ CD4^+^ T cells, resulting in a wide cancer cell apoptosis, significant tumor growth inhibition, and prolonged host survival [91]. Hence, the combined targeting of IL-30, in both CSCs and their environment, results in the combined decline of Tregs and myeloid cell populations in the TME, and amplifies the CTL effector functions.

The question of whether the immunological equilibrium resulting from the absence of IL-30 production can be ascribed to IL-30 in itself or to the co-existing lack of IL-27 or other p28-containing molecular complexes, remains a critical issue. However, the selective IL-30 blockade in PC–SLCs, which are unable to release detectable EBI3 and IL-27, led to a considerable anti-tumor efficacy with a clear implication for myeloid cells, whose ability to home to the tumor site is dramatically lost.

Importantly, the experimental results are consistent with the immuno- and clinical–pathological features of a cohort of more than one hundred patients who underwent surgery for PC. Analyses of clinical samples from PC patients with a high-grade (Gleason 8-10) and locally advanced disease (pT3N0M0), revealed that those with IL-30^−/−^PC, devoid of IL-30 in both cancer and immune cells, showed distinct TIA-1^+^ CD4^+^ CTLs, rare Tregs, and a lower biochemical recurrence rate, compared to patients with IL-30^+/+^PC, showing IL-30 expression in both cancer and infiltrating immune cells [91].

Targeting IL-30 in the TME, besides directly affecting cancer cell viability, subverts the cancer progression program and weakens the complicity between cancer and myeloid cells, providing a valuable tool to improve the response to a T cell-based immunotherapy that is customizable on patients with IL-30-expressing malignancies. These tumors have been estimated to represent 41% of the PC cases that have metastasized to the regional LNs, although the number of PCs harboring IL-30-producing myeloid cells exceeds 70% of metastatic cases [15].

## 5. Concluding Remarks and Future Directions

Immunotherapy, in particular checkpoint inhibitors, has led to an epoch-making progress in the treatment of advanced cancers [96,97]. However, there is a pressing need to improve its potential, especially for the cure of aggressive tumors that lack targetable molecules, such as TNBC [98], or tumors such as PC, which have demonstrated poor responsiveness in clinical trials [99,100].

The immunoregulatory molecule, IL-30, which has been recently identified in the context of the TME, specifically in BCs and PCs, has proven to mediate, directly or through second-level cytokines, the crosstalk between cancer and myeloid cells and to foster the cancer stem cell niche, thus, promoting tumor immune escape and disease progression [15,16,81]. Studies performed on immuno-competent murine models, xenotransplants and clinical samples, strongly suggest blocking of IL-30, produced by cancer and myeloid cells in the tumor and draining LNs, as a strategy to boost immunotherapy, in selected clinical cases.

Determination of the translational impact of experimental findings will speed-up the process to achieve this goal. Of paramount importance would be an unravelling of the effects of cancer- and immune cell-derived IL-30 on the different components of the tumor and lymphoid tissues, to determine the production of functional IL-30 in human tumors and to validate a sensitive and cost-effective method to assess the cytokine levels.

A two side targeting strategy that neutralizes cancer and immune cell-derived IL-30 might be entrusted to nanoscale delivery systems, due to their potential for targeting and minimizing off-target toxicity, while enhancing efficacy [101,102]. The multifunctionality of tumor targeting nanocarriers, that can incorporate gene therapy and targeted small molecules or immunotherapies [103], satisfies the need for cancer-selective checkpoint blockades and a simultaneous knockout of the *IL-30* gene, leaving the finely tuned immunological functions of the cytokine and cytokine containing complexes unaltered. Development of non-invasive, personalized, and integrated anti-cancer immunotherapies provide the prospective improvement of patient well-being, while extending life expectancy and satisfying unmet needs in oncology.
